# Evaluation of normalization methods for predicting quantitative phenotypes in metagenomic data analysis

**DOI:** 10.3389/fgene.2024.1369628

**Published:** 2024-06-05

**Authors:** Beibei Wang, Yihui Luan

**Affiliations:** ^1^ Frontier Science Center for Nonlinear Expectations, Ministry of Education, Qingdao, China; ^2^ Research Center for Mathematics and Interdisciplinary Sciences, Shandong University, Qingdao, China; ^3^ School of Mathematics, Shandong University, Jinan, China

**Keywords:** metagenomic, phenotype prediction, machine learning, normalization, scaling, compositional data analysis, transformation, batch correction

## Abstract

Genotype-to-phenotype mapping is an essential problem in the current genomic era. While qualitative case-control predictions have received significant attention, less emphasis has been placed on predicting quantitative phenotypes. This emerging field holds great promise in revealing intricate connections between microbial communities and host health. However, the presence of heterogeneity in microbiome datasets poses a substantial challenge to the accuracy of predictions and undermines the reproducibility of models. To tackle this challenge, we investigated 22 normalization methods that aimed at removing heterogeneity across multiple datasets, conducted a comprehensive review of them, and evaluated their effectiveness in predicting quantitative phenotypes in three simulation scenarios and 31 real datasets. The results indicate that none of these methods demonstrate significant superiority in predicting quantitative phenotypes or attain a noteworthy reduction in Root Mean Squared Error (RMSE) of the predictions. Given the frequent occurrence of batch effects and the satisfactory performance of batch correction methods in predicting datasets affected by these effects, we strongly recommend utilizing batch correction methods as the initial step in predicting quantitative phenotypes. In summary, the performance of normalization methods in predicting metagenomic data remains a dynamic and ongoing research area. Our study contributes to this field by undertaking a comprehensive evaluation of diverse methods and offering valuable insights into their effectiveness in predicting quantitative phenotypes.

## 1 Introduction

Microorganisms, which exist in and around us, play a significant role in shaping our overall health and living environment (Wang et al., 2015; Al Khodor et al., 2017; Foo et al., 2017; Horve et al., 2020). The development of high-throughput next-generation sequencing (NGS) technologies has recently advanced the efficiency and cost-effectiveness of studying microbial communities. Understanding and characterizing these communities continue to be ongoing goals for numerous research organizations (Bouchie, 2016; Hadrich, 2020). Despite the transformative impact of NGS on microbiome research, analyzing microbiome data poses challenges such as compositionally, sparsity, and high variability, for which standard statistical methods may not always provide comprehensive solutions (Swift et al., 2023).

There has been a growing interest in statistical methods that address the challenges associated with microbiome data analysis over the past decade. The two primary steps in statistical analysis of microbiome data involve normalization, which aims to mitigate systematic variations and biases, and differential abundance analysis, which identifies microbes with significantly different abundances under distinct observational or experimental conditions. While normalization methods are primarily designed for other data types, such as batch mean centering (BMC) (Sims et al., 2008) and Combat (Johnson et al., 2007) for DNA microarray data, or trimmed mean of M-values (TMM) (Robinson and Oshlack, 2010) and relative log expression (RLE) (Anders and Huber, 2010) for RNA-Seq data, they can also be applied to microbiome data. Several studies have investigated the performance of various normalization methods in the differential analysis of microbiome data. However, the conclusions may differ according to the purpose of analysis. McKnight et al. (McKnight et al., 2019) found that total sum scaling (TSS) and Rarefaction enable more accurate comparisons between communities and are the only methods that effectively normalize for sequencing depth across samples. Conversely, McMurdie and Holmes (2014) and Weiss et al. (2017) demonstrated that rarefaction alone is insufficient for data normalization and may result in a loss of valuable information within the dataset.

The utilization of microbiome data to predict phenotypes has become increasingly important in the era of high-throughput sequencing and metagenomics. To enhance the reproducibility of predictive models in multi-omics, many studies have been dedicated to mitigating heterogeneity in predictions. These studies often involve merging data from distinct datasets into one and treating them as if they originate from the same dataset to improve prediction accuracy (Thomas et al., 2019; Wirbel et al., 2019). Alternatively, researchers integrate the trained predictors from different datasets using diverse strategies to generate enhanced predictions (Patil and Parmigiani, 2018; Zhang et al., 2021). The potential contributions of normalization methods in prediction are primarily focused on DNA microarray or RNA-Seq data (Zwiener et al., 2014; Franks et al., 2018). It is noteworthy that, unlike differential analysis, the primary aim of normalization methods in prediction is to reduce heterogeneity between the training and unknown testing data. Therefore, group-wise normalization methods like percentile normalization (PN) (Gibbons et al., 2018) and Wrench (Kumar et al., 2018) cannot be applied to prediction. Therefore, there is a need to systematically evaluate the prediction performance of normalization methods in prediction using microbiome data.

In our previous study (Wang et al., 2024), we evaluated twenty-two existing normalization methods and assessed their efficacy in predicting binary phenotypes using microbiome data. However, there has been comparatively less emphasis on predicting quantitative phenotypes, which include numerical and continuous traits such as Body Mass Index (BMI) or blood glucose levels. The prediction framework for quantitative phenotypes is currently receiving increasing attention due to its significance. For instance, Yun et al. (Yun et al., 2017) identified distinct differences in gut microbiome composition among individuals with varying BMIs, providing valuable insights into the influence of microbial communities on body weight. In another study, Krisko et al. (Krisko et al., 2020) suggested that the gut microbiome plays a role in regulating blood glucose levels, presenting opportunities for personalized interventions and treatments. Therefore, exploring the associations between the microbiome and quantitative health-related phenotypes is essential for unraveling the intricate interplay between the microbiome and human health, an area that has not been well addressed.

In this study, we examine the effects of heterogeneity on predicting quantitative phenotypes and aim to assess the performance of various normalization methods in predicting quantitative phenotypes across studies. To conduct this investigation, we utilized a diverse and extensive dataset comprising 31 shotgun sequencing datasets obtained from healthy stool samples. Each dataset was paired with a separate dataset for training and testing purposes separately, allowing for a thorough evaluation of prediction performance. We used the Root Mean Squared Error (RMSE) as the primary performance metric, given its significance in quantifying prediction accuracy. Additionally, we supplemented our analysis with simulation studies that address three types of heterogeneity: background distributions of taxa in populations, batch effects across studies from the same population, and phenotype-associated models in different studies. These simulations enabled us to evaluate the performance of normalization methods in controlled settings, yielding valuable insights into how they perform under different scenarios.

This study aims to inform researchers of the necessary knowledge to make informed decisions when analyzing metagenomic data. Ultimately, this research aims to improve the reliability and accuracy of predictions obtained from metagenomic datasets, advancing our understanding of the complex relationships between microbial communities and host phenotypes.

## 2 Materials and methods

### 2.1 Workflow for quantitative phenotype prediction using simulated or real metagenomic datasets

To investigate the performance of different normalization methods in cross-study quantitative phenotype predictions, we developed a comprehensive workflow based on the methodology for case-control phenotype prediction from our previous study (Wang et al., 2024). The workflow consists of four main stages: real data, simulation, normalization, and prediction.

**FIGURE 1:**
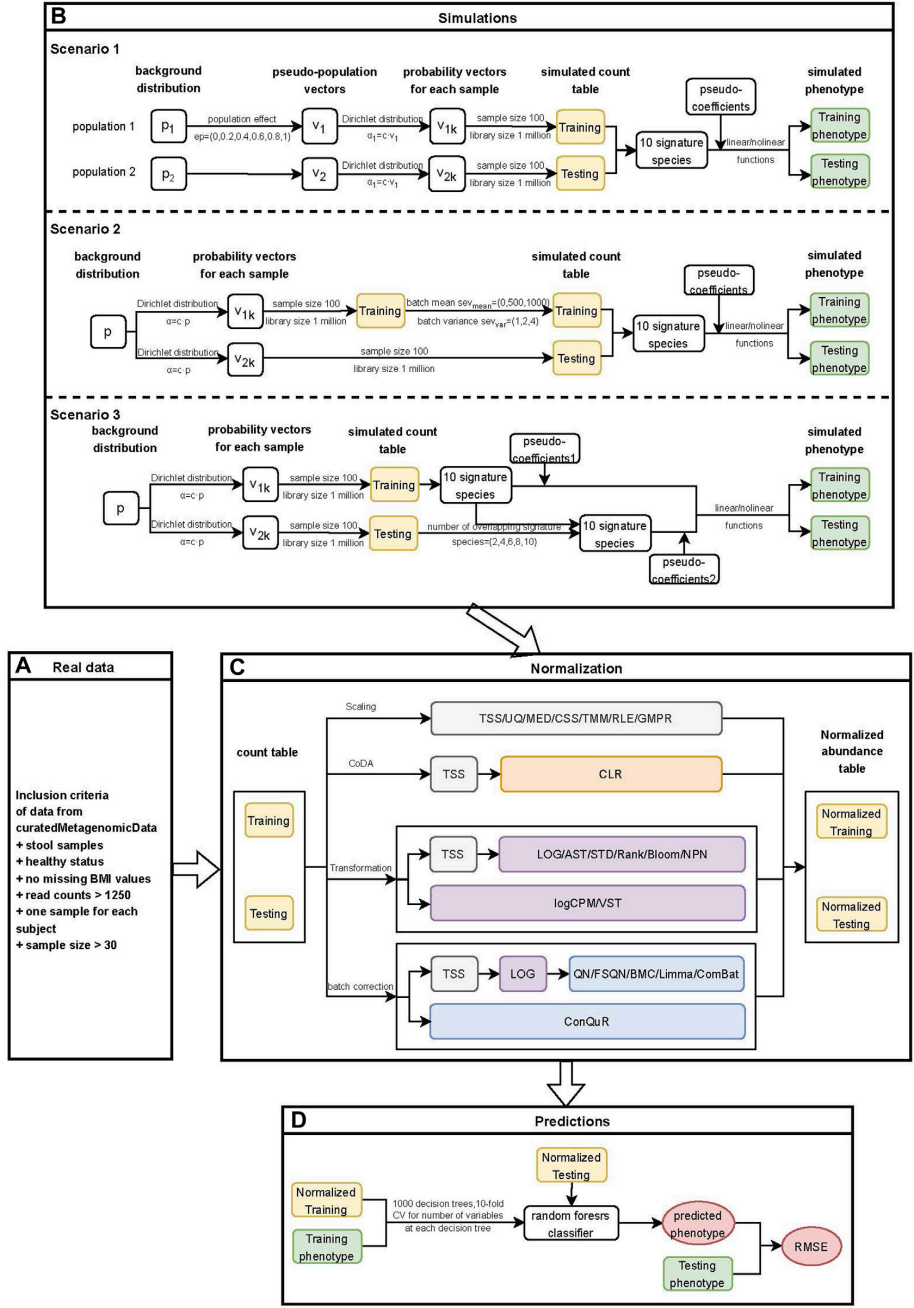
Workflow for quantitative phenotype prediction based on abundance profiles normalized by different methods. **(A)** Inclusion criteria for filtering data from curatedMetagenomicData. **(B)** Simulation stage of three different heterogeneity scenarios. Scenario 1: Different background distributions of taxa in populations. Scenario 2: Different batch effects of studies with the same background distribution of taxa in populations. Scenario 3: Different phenotype models of studies with the same background distribution of taxa in populations. The outputs from this step consisted of simulated count tables and simulated phenotypes of training and testing datasets. **(C)** Normalization stage. Twenty-two normalization methods were applied to both the real data and simulated data. The outputs from this step included normalized abundance tables of training and testing datasets. **(D)** Prediction stage. The outputs from the previous stage were used to train machine learning models, and the RMSE values of prediction models based on different normalization methods were further compared.

In the real data stage (Figure 1A), we selected samples from curatedMetagenomicData based on the inclusion criteria described in Section 2.2. The heterogeneity among different studies was examined using the statistical analysis methods outlined in Section 2.3. The cross-study predictions were performed by designating one dataset as the training set and choosing another from the remaining datasets as the testing set.

In the simulation stage (Figure 1B), we conducted three different scenarios. Firstly, we investigated the impact of different background distributions of taxa on quantitative phenotype predictions. To evaluate the performance of different normalization methods, we simulated two populations with distinct background distributions of taxa and then designated one population as the training set and the other as the testing set. Further details on the simulation of this scenario can be found in Section 2.4.1. Secondly, we simulated batch effects between training and testing datasets with varying severity levels. It is essential to address batch effects before conducting downstream analysis as they could compromise the reproducibility of genetic findings (Kupfer et al., 2012). We examined the influence of batch effects on quantitative phenotype predictions and provided a detailed description of the method in Section 2.4.2. Lastly, we investigated the impact of different underlying phenotype-associated feature models between the training and testing datasets on phenotype predictions. We assumed that the phenotype-associated features in the training and testing datasets would not be exactly the same and adjusted the number of overlapping features between them. Further details on this scenario can be found in Section 2.4.3.

In the normalization stage (Figure 1C), we employed various normalization methods to reduce heterogeneity within and across the real or simulated training and testing datasets. A total of 22 normalization methods were implemented, and detailed information about these methods was provided in Section 2.5. For scaling methods that involved the selection of references, such as TMM and RLE, as well as transformation methods that ensured prediction covariates (taxa) were drawn from the same distribution, including STD, Rank, Blom, NPN, and VST, we first normalized the training data. Subsequently, we combined the testing data with the training data and performed normalization on the combined dataset. The normalized testing data was obtained from the normalized combined data. This approach ensures the independence of the normalization process between the training and testing data while minimizing the heterogeneity between them (Warnat-Herresthal et al., 2020).

In the prediction stage (Figure 1D), we employed the random forest model to train the normalized training data and validate it using the normalized testing data. The performance of various normalization methods was evaluated using the RMSE. Additional details regarding this stage can be found in Section 2.6.

### 2.2 curatedMetagenomicData 3.8.0

The curatedMetagenomicData 3.8.0 package presented a curated meta-dataset of the human microbiome, derived from a collection of 93 cohorts involving shotgun sequencing of six distinct body sites. The raw sequencing data underwent a rigorous and standardized processing pipeline. Each sample in this dataset includes six primary data categories: gene family, marker abundance, marker presence, pathway abundance, pathway coverage, and relative taxonomic abundance values. Taxonomic abundance values were determined using MetaPhlAn3 (Beghini et al., 2021), while the assessment of metabolic functional potential was performed through HUMAnN3 (Franzosa et al., 2018). The package also provides curated clinical and phenotypic metadata. For more comprehensive insights, please refer to the official documentation of the curatedMetagenomicData package (Pasolli et al., 2017).

In order to compare the predictive performance of different methods for normalizing microbiome profiles in predicting BMI values, our analysis focuses specifically on healthy subjects obtained from the curatedMetagenomicData dataset. We selected subjects from all cohorts based on the following inclusion criteria: 1) stool samples; 2) healthy status; 3) no missing BMI values; 4) read counts exceeding 1,250. Additionally, if multiple samples were available for a subject, we randomly selected one for our analysis. We only included datasets with a sample size greater than 30. Supplementary Figure S1A shows the above inclusion criteria for filtering data from curatedMetagenomicData. In total, our analysis involved 5,963 samples from 31 datasets. Table 1 presents the characteristics of the curatedMetagenomicData datasets used in our analysis. We obtained the species count tables from these datasets and included them in the subsequent analysis.

**TABLE 1:** Characteristics of curatedMetagenomicData datasets involved in our analysis, including country, sample size, number of species in each dataset (No. Of species), DNA extraction kits (DNA-Exk), and sequencing platforms (Seq-Plat).

Dataset	Country	Sample size	No. Of species	DNA-Exk	Seq-plat
AsnicarF_2021 (Asnicar et al., 2021)	United States, United Kingdom	1,097	638	PowerSoilPro	IlluminaNovaSeq
CosteaPI_2017 (Costea et al., 2017)	Kazakhstan	84	437	Gnome	IlluminaHiSeq
DeFilippisF_2019 (De Filippis et al., 2019)	Italy	97	459	PowerSoil	IlluminaNextSeq
DhakanDB_2019 (Dhakan et al., 2019)	India	107	297	Qiagen	IlluminaNextSeq
HansenLBS_2018 (Hansen et al., 2018)	Denmark	58	354	NA	IlluminaHiSeq
HMP_2012 (The Human Microbiome Project Consortium, 2012)	United States	95	419	Qiagen	IlluminaHiSeq
JieZ_2017 (Jie et al., 2017)	China	164	538	Qiagen	IlluminaHiSeq
KarlssonFH_2013 (Karlsson et al., 2013)	Sweden, Germany, France, Iceland	43	335	NA	IlluminaHiSeq
KaurK_2020 (Kaur et al., 2020)	India	31	262	ZR_Fecal_DNA_MiniPrep	IlluminaHiSeq
KeohaneDM_2020 (Keohane et al., 2020)	Ireland	116	378	NA	IlluminaHiSeq
LeChatelierE_2013 (Le Chatelier et al., 2013)	Denmark	115	445	NA	IlluminaHiSeq
LifeLinesDeep_2016 (Zhernakova et al., 2016)	Netherlands	1,135	647	Qiagen	IlluminaHiSeq
LokmerA_2019 (Lokmer et al., 2019)	Cameroon	56	381	Illuminakit	IlluminaHiSeq
NagySzakalD_2017 (Nagy-Szakal et al., 2017)	United States	50	366	KAMA_Hyper_Prep	IlluminaHiSeq
NielsenHB_2014 (Nielsen et al., 2014)	Spain	59	404	NA	IlluminaHiSeq
Obregon-TitoAJ_2015 (Obregon-Tito et al., 2015)	Peru/United States	51	387	MoBio	IlluminaHiSeq
PasolliE_2019 (Pasolli et al., 2019)	Madagascar	112	446	Qiagen	IlluminaHiSeq
QinJ_2012 (Qin et al., 2012)	China	174	534	NA	IlluminaHiSeq
QinN_2014 (Qin et al., 2014)	China	114	443	NA	IlluminaHiSeq
RubelMA_2020 (Rubel et al., 2020)	Cameroon	86	334	PSP_Spin_Stool	IlluminaHiSeq
SchirmerM_2016 (Schirmer et al., 2016)	Netherlands	456	490	Illuminakit	IlluminaHiSeq
ThomasAM_2018 (Thomas et al., 2019)	Italy	39	393	Qiagen/Gnome	IlluminaHiSeq
VogtmannE_2016 (Vogtmann et al., 2016)	United States	52	423	Gnome	IlluminaHiSeq
WirbelJ_2018 (Wirbel et al., 2019)	Germany	65	385	Gnome	IlluminaHiSeq
XieH_2016 (Xie et al., 2016)	United Kingdom	169	537	Qiagen	IlluminaHiSeq
YachidaS_2019 (Yachida et al., 2019)	JPN	245	604	NA	IlluminaHiSeq
YeZ_2018 (Ye et al., 2018)	China	45	305	Qiagen	IlluminaHiSeq
YuJ_2015 (Yu et al., 2017)	China	38	403	Qiagen	IlluminaHiSeq
ZeeviD_2015 (Zeevi et al., 2015)	Israel	870	673	NA	IlluminaHiSeq
ZellerG_2014 (Zeller et al., 2014)	France	59	515	Gnome	IlluminaHiSeq
ZhuF_2020 (Zhu et al., 2020)	China	81	402	NA	IlluminaHiSeq

### 2.3 Statistical analysis

We performed microbial relative abundance calculations for each sample and computed the Shannon indices using the *diversity*() function from the R package *vegan* (Oksanen et al., 2007). The differences in Shannon indices between each dataset and the overall Shannon indices were determined using the Wilcoxon rank sum test. The dissimilarities between sample pairs were quantified using the Bray-Curtis distance (Bray and Curtis, 1957), implemented by the *vegdist*() function from the R package *vegan* (Oksanen et al., 2007). Principal coordinate analysis (PCoA) was employed to effectively visualize the sample clustering, using the *pcoa*() function from the R package *ape* (Paradis and Schliep, 2019). To assess the variance attributable to population factors, we conducted permutational multivariate analysis of variance (PERMANOVA) (Anderson, 2001) using the *adonis*() function in the R package *vegan* (Oksanen et al., 2007). To avoid issues with variable ordering, the total variance explained by each variable was evaluated independently of other variables, and thus should be regarded as the total variance explainable by that variable (Lloyd-Price et al., 2019).

### 2.4 Simulation study

In line with our previous investigation on case-control studies (Wang et al., 2024), we devised three unique scenarios to account for the heterogeneity within the training and testing data. For each combination of the parameters, we iterated the procedure 100 times. Subsequently, the datasets underwent normalization using various methods. Employing the random forest algorithm, we constructed prediction models based on one simulated population and evaluated their performance on the other population in each of the three scenarios. To assess the accuracy of the predictions, we computed the RMSE values for the 100 simulation runs conducted across the different scenarios. The workflows of the simulation stage are presented in Figure 1B).

#### 2.4.1 Scenario 1: different background distributions of taxa in populations

In the first scenario, we considered that the variations between populations were attributable to differences in the underlying distributions of taxa, such as ethnicity or diet. McMurdie and Holmes (McMurdie and Holmes, 2014) proposed a method to simulate samples from distinct populations (Simulation A) and samples with case-control designs (Simulation B) independently within this particular scenario. In our simulations, we combined these strategies and implemented specific modifications.

Our methodology commenced by establishing the baseline levels of taxon abundance for the training and testing populations. To replicate this scenario, we collected two publicly available and geographically diverse datasets, GuptaA_2019 and FengQ_2015. The control samples from these two datasets were used as the template in our simulations, which is the same as our previous analysis (Wang et al., 2024). Specifically, we included 30 control samples and 183 species from the GuptaA_2019 dataset (Dhakan et al., 2019; Gupta et al., 2019) for training purposes, and 61 healthy samples and 468 species from the FengQ_2015 dataset (Feng et al., 2015) for testing purposes. For each dataset, a count table consisting of rows representing taxa and columns representing samples was povided. By summing the rows, we obtained the initial vectors representing the underlying taxon abundance in different populations, denoted as *p*_*k*_, where *k* = 1, 2.

To explore the influence of dissimilarities between two populations on cross-study prediction, we constructed pseudo-population vectors *v*_*k*_, where *k* = 1, 2:

$$v_{1}=ep\cdot p_{1}+\left(1-ep\right)\cdot p_{2},\;v_{2}=p_{2},$$ (1)

where *ep* denotes the population effect that quantifies the differences between two populations. It should be emphasized that $v_{1}^{\prime }-v_{2}^{\prime }=ep\left(v_{1}-v_{2}\right)$ , which underscores how the differences between the two simulated populations escalated with increasing values of *ep*. By incrementally varying *ep* from 0 to one in intervals of 0.2, we analyzed the overall trends of different normalization methods.

To generate pseudo read counts for 100 samples within each population, we assumed that the taxonomic probabilities *x*_*kj*_ of sample *j* belonging to population *k* followed a Dirichlet distribution *Dir* (*α*_*k*_), with *α*_*k*_ = *c* ⋅ *v*_*k*_ for *k* = 1, 2. To ensure minimal variation, we assigned a large value to *c*, resulting in a variance of *x*_*kj*_ that approximates 0 and aligns with *v*_*k*_. To introduce some level of variability, we selected *c* = 1 × 10^6^ (preventing the generation of zero probabilities). The read counts for each sample were simulated using a multinomial distribution *MN*(library size, *x*_*kj*_), *k* = 1, 2, where the library size was set to 1,000,000 and the probabilities were derived from the Dirichlet distribution.

Among the 154 taxa shared by the two populations, we randomly chose 10 taxa and proposed that these taxa were linked to a specific quantitative phenotype of interest. It was assumed that the first five taxa exhibited enrichment while the remaining five were diminished. A vector of pseudo coefficients was generated from a uniform distribution with lower and upper bounds of three and five for positive associations, and −5 and −3 for negative associations. The chosen taxa and their corresponding pseudo coefficients remained consistent throughout the simulations. The quantitative phenotypes were simulated based on the relationship between the phenotype and the corresponding microbial abundances as follows.

• Linear: *y* = *c*_1_ *β* ^*T*^ *x* + *ϵ*
• Quadratic: *y* = *c*_2_ *β* ^*T*^ *x* ^2^ + *ϵ*
• Inverse: $y=\frac{c_{3}}{\beta^{T}x}+\epsilon$

• Logistic: $y=\frac{c_{4}}{1+\exp\left(\beta^{T}x\right)}+\epsilon$

Where *x* is the vector of the selected phenotype associated with microbial relative abundance, *β* indicates the pseudo coefficients, *c*_1_, *c*_2_, *c*_3_, *c*_4_ represents constants used to control the range of absolute values of the simulated phenotypes *y* (ranging in dozens or hundreds), and *ϵ* ∼ *N* (0, 1) represents random noise.

#### 2.4.2 Scenario 2: different batch effects of studies with the same background distribution of taxa in populations

In this scenario, we employed the controls in FengQ_2015 dataset (Feng et al., 2015) as the template for our simulations, ensuring that the background distribution remained consistent between the training and testing datasets. By doing so, we effectively eliminated the population effects observed in Scenario 1. The generation of read counts and phenotypes followed the same procedure as in Scenario 1, utilizing multinomial distributions with a sample size of one million reads. Specifically, we specified 10 taxa associated with the phenotype, and considered linear, quadratic, inverse, and logistic relationships between the phenotype and the corresponding microbial abundances.

To simulate batch effects, we followed a similar procedure as described in Zhang et al. (Zhang et al., 2021). We assumed that the mean (*γ*_*ik*_) and variance (*δ*_*ik*_) of taxon *i* were influenced by the batch *k*. Drawing from the batch effect generating model proposed by Johnson et al. (Johnson et al., 2007), we assumed an additive effect on the mean and a multiplicative effect on the variance for each taxon. The values of *γ*_*ik*_ and *δ*_*ik*_ were randomly sampled from normal and inverse gamma distributions, respectively, as expressed by:

$$\gamma_{ik}∼N\left(\mu_{k},\sigma_{k}^{2}\right),\;\delta_{ik}∼\text{InvGamma}\left(\alpha_{k},\beta_{k}\right).$$ (2)

To specify the hyperparameters (*μ*_*k*_, *σ*_*k*_, *α*_*k*_, *β*_*k*_), we defined two values to indicate the severity of batch effects. Specifically, we considered three levels for the batch effect on the mean (*sev*_*mean*_ ∈ {0, 500, 1,000}) and three levels for the batch effect on the variance (*sev*_var_ ∈ {1, 2, 4}). For a given severity level, the variance of *γ*_*ik*_ and *δ*_*ik*_ was fixed at 0.01, while the batch effect parameters were either added or multiplied to the mean and variance of the original study’s expression. Importantly, the batch effects were solely applied to the training data, while the test dataset remained unaltered.

#### 2.4.3 Scenario 3: different phenotype models of studies with the same background distribution of taxa in populations

In this scenario, we hypothesized that the model for phenotype-associated taxa may differ between populations. To mitigate the population effects mentioned in Scenario 1, we employed the FengQ_2015 dataset (Feng et al., 2015) as the template for simulations. In order to eliminate the batch effects described in Scenario 2, this simulation scenario did not incorporate any batch effects.

To select phenotype-associated taxa, we predetermined 10 taxa for the training data. From the initial 10 taxa, we selected a subset and added additional taxa to maintain a total of 10 signature taxa in the testing data. The level of resemblance between the training and testing data was determined by the number of taxa that overlapped, ranging from 2 to 10 with increments of 2. Subsequently, the two populations were simulated following the same procedure as in the previous two scenarios. The simulation parameters consisted of 100 samples per population, one million reads per sample, and four distinct relationships between quantitative phenotype and phenotype-associated taxa.

### 2.5 Normalization methods

Microbiome data analysis commonly employs a range of normalization methods. In predicting the quantitative traits of unknown samples, it is crucial to transform or normalize the data to ensure that both the training and testing datasets came from the same underlying distribution. This investigation encompassed a comprehensive comparative analysis, examining seven scaling methods, one approach based on compositional data analysis (CoDA), eight transformation methods, and six batch correction methods. To the best of our knowledge, this study represents the most thorough comparison conducted to date, focused on prediction.

Suppose we have a dataset with *n* samples and *m* features. Denote the count for taxon *i* in sample *j* as *c*_*ij*_. With this notation, the procedures and equations for normalization methods can be outlined as follows.

#### 2.5.1 Scaling methods

Scaling is a commonly used method to reduce biases introduced by sequencing technology. It is often sample-specific and is achieved by dividing the counts in a sample by a scaling factor. Mathematically, this can be represented by the following equation:

$$x_{ij}=\frac{c_{ij}}{s_{j}},$$ (3)

where *x*_*ij*_ is the normalized abundance for taxon *i* in sample *j*, and *s*_*j*_ is the scaling factor for sample *j*.

The Total Sum Scaling (TSS) method is the simplest scaling method used to correct for differences in sequencing depth (Dillies et al., 2013). It scales each sample by the total number of reads in that sample. Upper Quartile (UQ) (Bullard et al., 2010; Dillies et al., 2013) and Median (MED) (Dillies et al., 2013) are similar to TSS, except that they scale each sample by the upper quartile or the median of sample counts different from zero, rather than the total number of reads. Cumulative Sum Scaling (CSS) (Paulson et al., 2013) is a modification of TSS specifically designed for microbiome data. It computes the scaling factor as the cumulative sum of counts, up to a percentile $\hat{l}$ determined by the data. Trimmed Mean of M-values (TMM) (Robinson and Oshlack, 2010) and Relative Log Expression (RLE) (Anders and Huber, 2010) are commonly used normalization methods for RNA-Seq data with the assumption that most genes are not differentially expressed. TMM first selects a reference sample, and all other samples are compared to this reference. The TMM size factor is then calculated as the weighted trimmed mean of the log ratios. RLE, on the other hand, calculates the geometric mean of all genes as a reference, and each sample is compared to this reference to generate ratios (fold changes) for all genes. The RLE size factor is obtained by taking the median of these ratios. The Geometric Mean of Pairwise Ratios (GMPR) (Chen L. et al., 2018) extends the concept of RLE normalization by reversing the order of computing the geometric mean and the median. This extension overcomes the zero-inflation issue frequently observed in microbiome data.

All the scaling methods were directly applied to the microbial count data and the workflows of scaling methods were shown in Figure 1C). The formulas for the scaling factors used in our analysis are presented in Table 2.

**TABLE 2:** Summary of scaling methods.

Method	Scaling factor	Scaling factor/method description	Data designed for	Availability (bioconductor/R)
TSS	*s*_*j*_ = *∑*_*i*_ *c*_*ij*_	Total number of sample reads	None	stats
UQ	*s*_*j*_ = *q* ^3^(*P*_*j*_)	Upper quartile of sample counts different from 0	RNA-Seq	stats
MED	$s_{j}=\text{Median}\left(P_{j}\right)$	Median of sample counts different from 0	RNA-Seq	stats
CSS	$s_{j}=\frac{\sum_{i|i\in M_{j}}c_{ij}}{N^{\text{CSS}}}$	Cumulative sum of counts (up to a percentile $\hat{l}$ determined by the data)	microbiome	metagenomeSeq (Paulson et al., 2013)
TMM	$\log_{2}\left(s_{j}\right)=\frac{\sum_{i\in m_{jk}^{\text{TMM}}}\left(w_{jk}^{i}M_{jk}^{i}\right)}{\sum_{i\in m_{jk}^{\text{TMM}}}\left(w_{jk}^{i}\right)}$	Trimmed mean of log-ratios	RNA-Seq	edgeR (Robinson et al., 2010)
RLE	$s_{j}={\text{Median}}_{i}\left\{\frac{c_{ij}}{G\left(c_{i}\right)}\right\}$	Median fold-change relative to a References	RNA-Seq	DESeq2 (Love et al., 2014)
GMPR	$s_{j}={\left(\prod_{j}{\text{Median}}_{i|c_{ij}\cdot c_{ik}\neq 0}\left\{\frac{c_{ij}}{c_{ik}}\right\}\right)}^{\frac{1}{m}}$	Geometric mean of ratios between pairs of samples	microbiome	GUniFrac (Chen et al., 2018a)

*q* ^3^ (⋅) is the function of estimating upper quartile; Median (⋅) is the function of estimating median; *P*_*j*_ = {*c*_*ij*_|*c*_*ij*_ > 0, *i* = 1, … , *n*} represents a set of counts different from 0 in sample *j*; $M_{j}=\left\{c_{ij}|c_{ij}\leq q_{\hat{l}}\left(c_{j}\right)\right\}$ denotes the taxa included in the cumulative summation for sample *j*; *N* ^CSS^ is an appropriately chosen normalization constant; M-values $M_{jk}^{i}=\log_{2}\frac{c_{ij}/\sum_{i}c_{ij}}{c_{ik}/\sum_{i}c_{ik}}$ is the log2 of the ratio of two observed relative abundance for a taxon *i*; A-values $A_{jk}^{i}=\frac{1}{2}\log_{2}\left(\frac{c_{ij}}{\sum_{i}c_{ij}}\frac{c_{ik}}{\sum_{i}c_{ik}}\right)$ is the log2 of the geometric mean of the observed relative abundance; $m_{jk}^{\text{TMM}}$ is the remaining taxa after the trimming M-values by 30% and the A-values by 5%; $w_{jk}^{i}=\frac{\sum_{i}c_{ij}-c_{ij}}{c_{ij}\sum_{i}c_{ij}}+\frac{\sum_{i}c_{ik}-c_{ik}}{c_{ik}\sum_{i}c_{ik}}$ represents the weight; $G\left(c_{i}\right)={\left(\prod_{j=1}^{m}c_{ij}\right)}^{\frac{1}{m}}$ is the geometric mean of gene *i*.

#### 2.5.2 Compositional data analysis (CoDA) methods

High-throughput sequencing microbiome datasets are compositional due to the arbitrary total imposed by the sequencing instrument (Gloor et al., 2017). The collection of methods used to analyze compositional data is compositional data analysis (CoDA) introduced by Aitchison et al. (Aitchison, 1982). They mitigate the impact of sampling fractions by converting the abundances into log ratios within each sample. The most known log-ratio transformation is centered log-ratio transformation (CLR) (Aitchison, 1982). It calculates the log-ratio of counts and their geometric means within each sample based on relative abundances. Two other transformations that are sometimes used in CoDA are additive log-ratio (ALR) (Aitchison, 1982) and isometric log-ratio (ILR) (Aitchison, 1982). Both of them use a single component as a reference. However, the choice of reference proposes computational challenges arising from the large number of taxa. As a result, our analysis solely focused on CLR. Another limitation of log-ratio transformations is that they do not account for zeros. We add a pseudo count of 0.65 times the minimum non-zero abundance to the zero values (Martín-Fernández et al., 2003).

The TSS normalized data are still compositional since the total sum of abundances for a sample is fixed to 1. To address the sample-specific differences, we applied the frequently used TSS normalization prior to performing the CLR transformation. The workflow for the CLR can be found in Figure 1C). The formula for the CLR transformation is provided in Table 3.

**TABLE 3:** Summary of transformation methods.

Methods	Transformation	Preprocess	Adjustment	Data designed for	Availability (bioconductor/R)
CLR	$\log\frac{x_{ij}}{G\left(x_{j}\right)}$	TSS		compositional data	compositions (Van den Boogaart and Tolosana-Delgado, 2008)
LOG	log *x*_*ij*_	TSS	Skewness	—	stats
AST	$\arcsin\sqrt{x_{ij}}$	TSS	Skewness, Extreme values	—	stats
STD	$\frac{x_{ij}-\mu_{i}}{\sigma_{i}}$	TSS	Unequal variances	—	stats
Rank	*r*_*ij*_	TSS	Skewness, Extreme values, Unequal variances	RNA-Seq	stats
Blom	$\Phi^{-1}\left(\frac{r_{ij}-c}{m+1}\right)$	TSS	Skewness, Extreme values, Unequal variances	RNA-Seq	stats
NPN	$\Phi^{-1}\left(\delta \right),\text{ if }{\hat{r}}_{ij}\leq \delta$ $\Phi^{-1}\left({\hat{r}}_{ij}\right),\text{ if }\delta <{\hat{r}}_{ij}\leq 1-\delta$ $\Phi^{-1}\left(1-\delta \right),\text{ if }{\hat{r}}_{ij}\geq 1-\delta$	TSS	Skewness, Extreme values, Unequal variances	—	huge (Jiang et al., 2021)
logCPM	$\log_{2}\frac{c_{ij}}{10^{6}}$	None	Skewness	RNA-Seq	edgeR (Robinson et al., 2010)
VST	$\int_{0}^{c_{ij}}\frac{1}{v\left(\mu_{i}\right)}d\mu_{i}$	None	Skewness	RNA-Seq	DESeq2 (Love et al., 2014)

*C*_*ij*_ and *x*_*ij*_ represent the count and relative abundance of taxon *i* in sample *j*; $G\left(x_{j}\right)={\left(\prod_{i=1}^{n}x_{ij}\right)}^{\frac{1}{n}}$ is the geometric mean of sample *j*; *μ*_*i*_ and *σ*_*i*_ is the mean and standard deviation of taxon *i*; *r*_*ij*_ is the corresponding rank for relative abundance *x*_*ij*_; $c=\frac{3}{8}$ is a constant; Φ^−1^ (⋅) denotes the quantile function of normal distribution; ${\hat{r}}_{ij}=\frac{r_{ij}}{m+1}$ ; $\delta =\frac{1}{4m^{1/4}\sqrt{\pi \log m}}$ ; $v\left(\mu_{i}\right)=\sigma_{i}^{2}=\mu_{i}+a_{i}\mu_{i}^{2}$ , with $a_{i}=a_{0}+\frac{a_{1}}{\mu_{i}}$ being a dispersion parameter and *a*_0_ and *a*_1_ are estimated in a generalized linear model.

#### 2.5.3 Transformation methods

Microbiome data exhibit several problematic properties, including skewed distributions, unequal variances for individual taxa, and extreme values. To address these issues when fitting the prediction model, we proposed to apply transformations to the microbiome data. These transformations can address one, two, or all of these problems. We investigated the influence of eight popular transformation methods in prediction, including LOG, arcsine square-root (AST), standardization (STD), rank, blom, non-paranormal (NPN), log counts per million (logCPM), and variance stabilizing transformation (VST).

The log transformation is commonly used to address skewed distributions of taxa abundances, resulting in transformed abundances that are closer to a normal distribution (Zwiener et al., 2014). To prevent infinite values, a pseudo count of 0.65 times the minimum non-zero abundance is added to the zero values (Martín-Fernández et al., 2003). Another method, AST, is employed to reduce the occurrence of extreme values in the data and achieve a more approximately normal distribution. STD is the default implementation in many regression analyses for reducing variations in input features. Rank transformation (Zwiener et al., 2014), widely used in non-parametric statistics, ensures that the transformed features are uniformly distributed between zero and the sample size *m*. In order to handle ties in zero counts, a small noise term *ϵ*_*ij*_ ∼ *N* (0, 10^–10^) is added before the data transformation. Blom transformation (Beasley et al., 2009; Zwiener et al., 2014) further takes the uniformly distributed ranks and converts them into a standard normal distribution. The non-paranormal (NPN) transformation (Liu et al., 2009) initially converts variables into univariate smooth functions to estimate a Gaussian copula but can also be used independently for analysis purposes. Log counts per million (logCPM) is a descriptive measure used to assess gene expression levels in RNA-Seq data. In our analysis, we applied this transformation to the microbiome data by adding a pseudo count equal to 0.65 times the minimum non-zero abundance to the zero values before performing the logarithmic transformation (Martín-Fernández et al., 2003). The Variance Stabilizing Transformation (VST) (Anders and Huber, 2010) models the relationship between mean and variance for each taxon. As a result, the variance-stabilized counts exhibit a less skewed distribution but may contain many extreme values. A pseudo count one was added to zero values as integer inputs are needed when realizing VST. The formulas for the eight transformation methods examined in our analysis can be found in Table 3.

**FIGURE 2:**
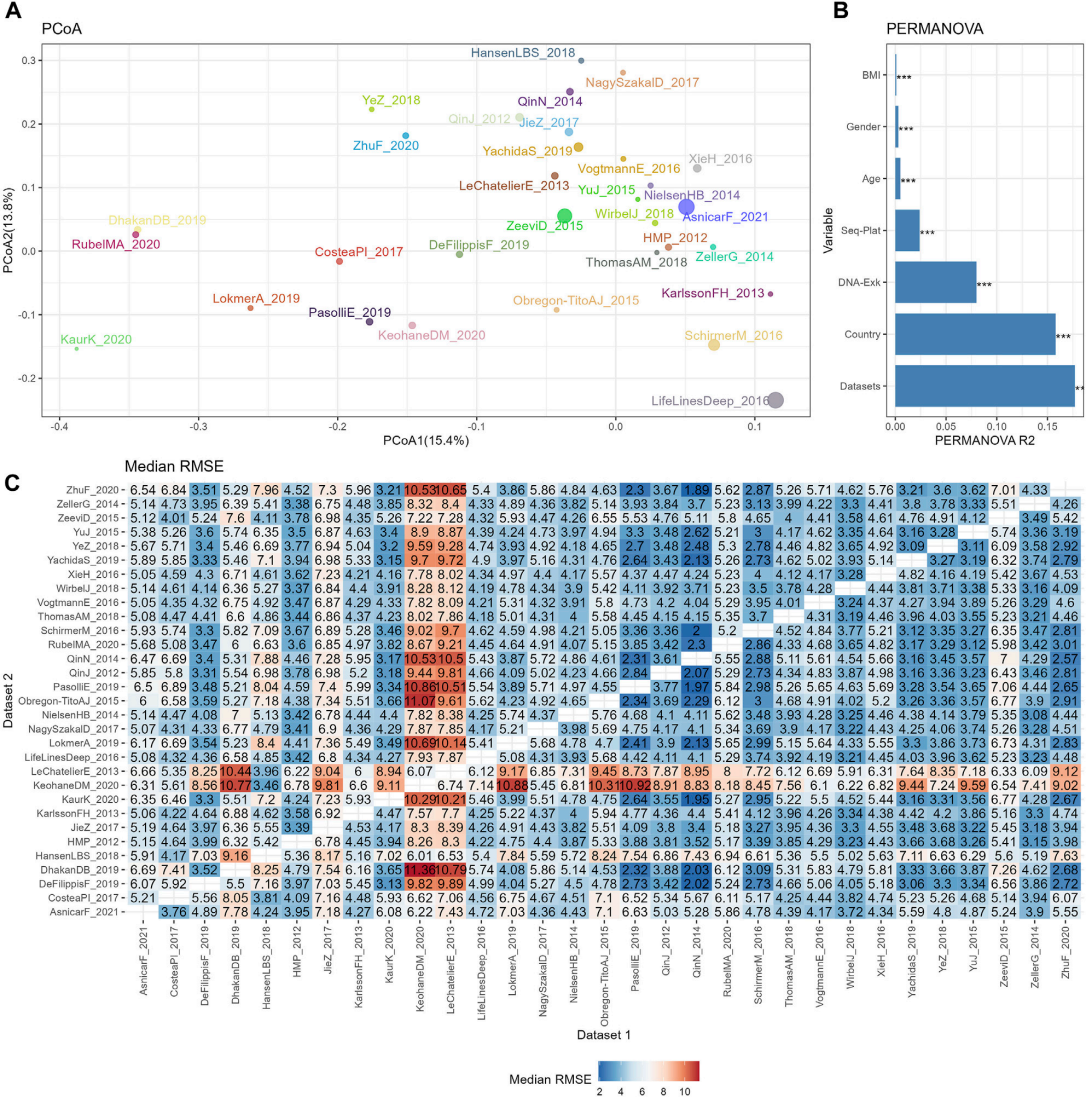
The reproducibility of cross-datasets prediction is limited by various confounding variables. **(A)** PCoA plot based on Bray-Curtis of TSS normalized abundances, with colors for different datasets and sizes for different sample sizes. **(B)** Bar plot of variance explained by different variables (R2) from PERMANOVA based on Bray-Curtis distance. *p* values calculated by 1,000 permutations were annotated on the top of the bar, with *** for *p* <0.001. **(C)** Median RMSE of cross-datasets prediction based on TSS normalized abundances using random forests model over 10 repetitions.

**FIGURE 3:**
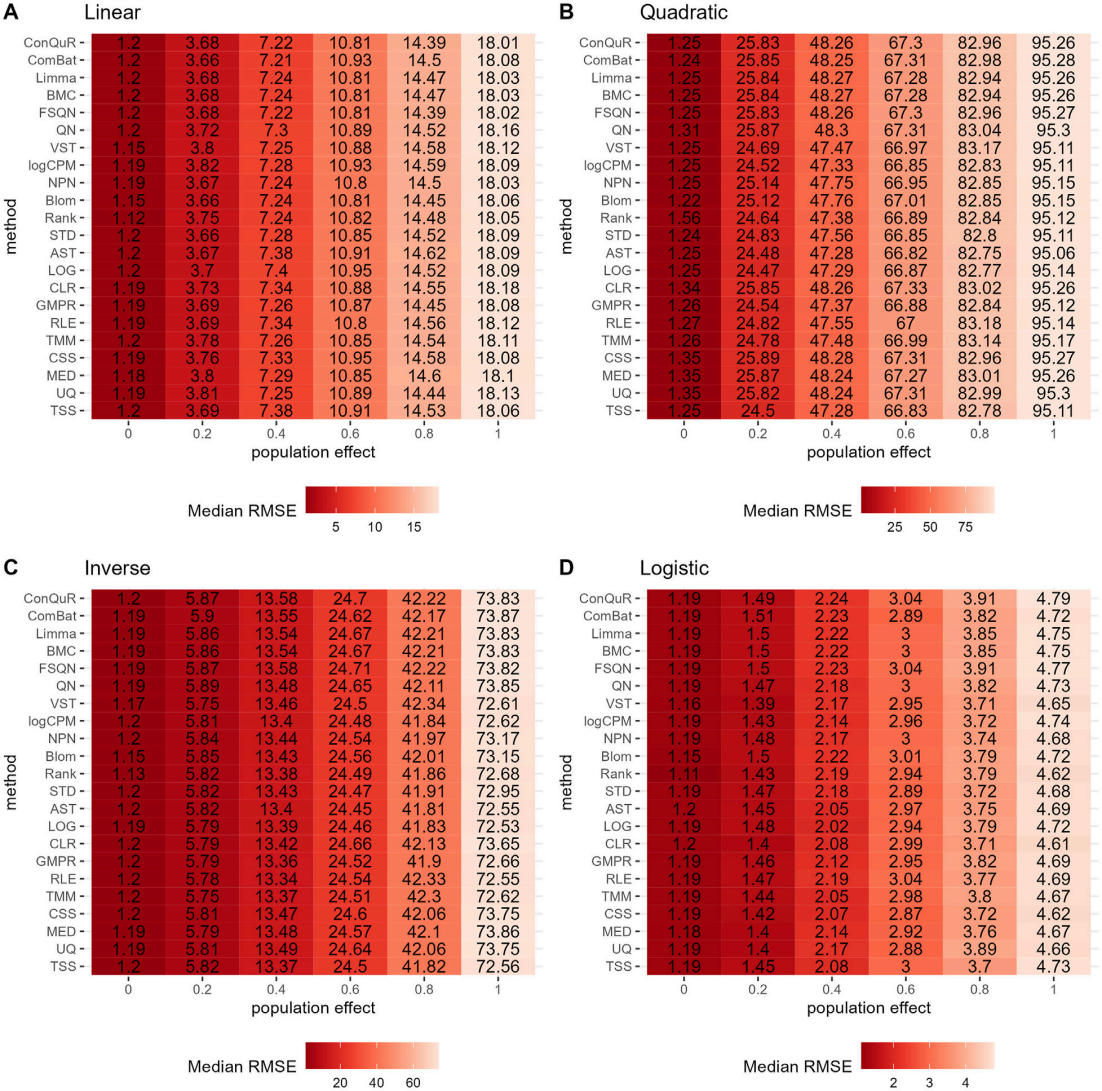
Heatmaps depicting median RMSE values obtained from abundance profiles normalized by different methods for predicting simulated quantitative phenotype in Scenario 1. The panels correspond to relationships between phenotype and phenotype-associated taxa, including **(A)** linear, **(B)** quadratic, **(C)** inverse, and **(D)** logistic. The columns represent different values of population effects, while the rows represent different normalization methods.

**FIGURE 4:**
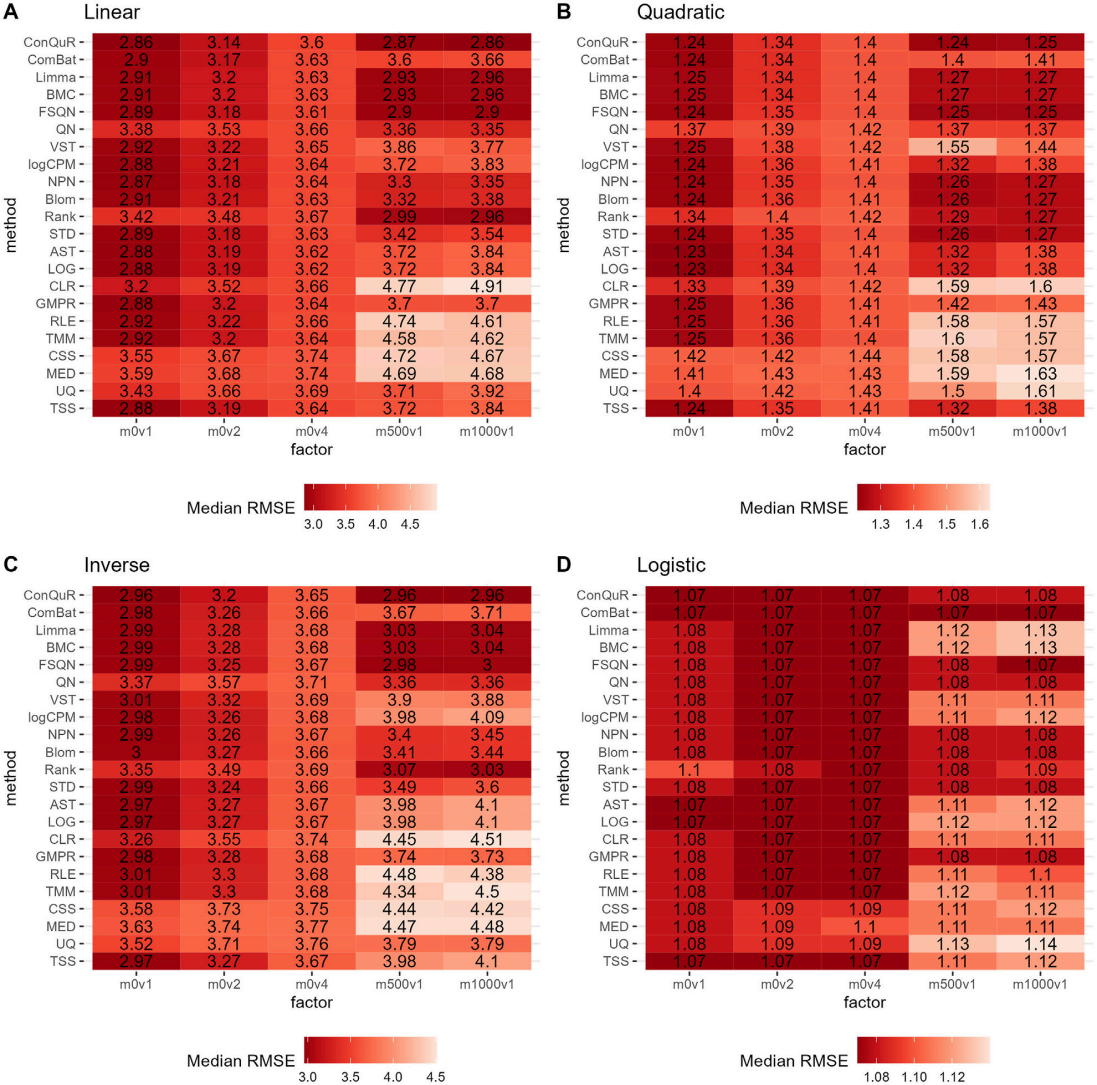
Heatmaps depicting median RMSE values obtained from abundance profiles normalized by different methods for predicting simulated quantitative phenotype in Scenario 2. The panels correspond to relationships between phenotype and phenotype-associated taxa, including **(A)** linear, **(B)** quadratic, **(C)** inverse, and **(D)** logistic. The columns represent different combinations of batch mean and batch variation, with “m” for batch mean adjusting the mean and “v” for batch variance adjusting the variance. The rows represent different normalization methods.

The transformation methods mentioned above are mostly feature-specific. In order to address the sample-specific differences, these methods are typically combined with scaling methods, with the exception of logCPM and VST. Based on the comparable performances of scaling methods in quantitative phenotype prediction, as demonstrated in Figures 2–4, we opted to apply transformations solely to the simplest and widely adopted method, the TSS normalized abundance. The workflows of the transformation methods are depicted in Figure 1C).

#### 2.5.4 Batch correction methods

Batch effects frequently occur in genomic technologies and can result from various specimen processing steps. Normalization methods alone may not adequately address these batch effects (Zhang et al., 2020). The differences in the overall expression distribution of each sample across batches may be corrected by scaling methods such as TMM or RLE. However, the batch effects in composition cannot be fully corrected with normalization. Many approaches have been proposed to effectively remove batch effects for microarray or RNA-Seq data. We applied them to microbiome data and examined their influence on quantitative phenotype prediction. In this study, we examined six commonly used methods: Quantile normalization (QN), Feature specific quantile normalization (FSQN), Batch mean centering (BMC), Linear models for microarray data (Limma), ComBat, and Conditional quantile regression (ConQuR). Table 4 summarizes the involved batch correction methods.

**TABLE 4:** Summary of batch correction methods.

Methods	Preprocess	Methods description	Data designed for	Availability (bioconductor/R)
QN	TSS, LOG	Equal the quantiles of the distributions across different samples	DNA microarray	preprocessCore (Bolstad, 2021)
FSQN	TSS, LOG	Equal the quantiles of the distributions across different features	RNA-Seq	FSQN (Franks et al., 2018)
BMC	TSS, LOG	Subtract the mean abundance of batch per feature	DNA microarray	pamr (Hastie et al., 2019)
Limma	TSS, LOG	Use linear model to remove batch effects	DNA microarray	limma (Ritchie et al., 2015)
Combat	TSS, LOG	Use empirical Bayes framework to remove batch effects	DNA microarray	sva (Leek et al., 2012)
ConQuR	None	Use conditional quantile regression to remove batch effects	microbiome	ConQuR (Ling et al., 2022)

The QN method (Bolstad et al., 2003) was originally designed for DNA microarrays but has been adapted for various types of data. This approach replaces each value in a target distribution with the corresponding value from a reference distribution based on their rank order. In our analysis, we applied quantile normalization to the training data and used the resulting normalized distribution as the reference for the test data (Thompson et al., 2016). Another variation of QN is FSQN method (Franks et al., 2018), which normalizes features instead of samples for RNA-Seq data. In FSQN, the reference distribution consists of the genes in the training set, while the target distribution consists of the genes in the testing set. Another method commonly employed for batch effects removal is BMC (Sims et al., 2008). This method centers the data on a batch-by-batch basis by subtracting the mean abundance per gene for each dataset from the individual gene abundance. Limma (Ritchie et al., 2015) is a popular statistical method extensively used in genomics. It utilizes linear models to eliminate batch effects. ComBat (Johnson et al., 2007), on the other hand, incorporates an empirical Bayes framework to estimate and remove batch effects while preserving the relevant biological variation. Finally, ConQuR (Ling et al., 2022) offers a batch effects removal approach that uses conditional quantile regression to deal with count tables.

The above-mentioned batch correction methods did not address the differences among samples. To account for this, we applied TSS method to normalize the training and testing data. Subsequently, we log-transformed the TSS-normalized abundance before applying the batch correction methods. To handle zero values, we replaced them with a pseudo relative abundance equivalent to 0.65 times the minimum non-zero abundance across the entire abundance table (Martín-Fernández et al., 2003). It is important to note that ConQuR, unlike the other methods, operates directly on microbial counts. The workflow of the batch correction methods is presented in Figure 1C) and a summary of the batch correction methods can be found in Table 4.

### 2.6 The random forest classifiers

The random forest algorithm is a supervised learning approach that is capable of handling both regression and classification problems (Liaw and Wiener, 2002). In our previous case-control study (Wang et al., 2024), we employed random forest classification to determine disease status, while in this study, we used random forest regression to predict a quantitative phenotype. The random forest algorithm is well known for reducing the overfitting problem with large numbers of predictors and handling complex, high-dimensional data characterized by non-linear relationships. Compared with SVM and LASSO, it has been shown to outperform them when applied to microbiome data (Pasolli et al., 2016). The implementation of random forest was carried out using the *train*() function from the R package *caret* (Kuhn, 2008). We constructed a random forest with 1,000 decision trees, and the number of variables at each decision tree was optimized through grid search using 10-fold cross-validation.

We evaluated the performance of our predictions using the RMSE, which quantifies the square root of the average squared differences between predicted and actual values. The RMSE is defined as follows:

$$RMSE=\sqrt{\frac{1}{n}\overset{n}{\underset{j=1}{\sum }}{\left(y_{\mathrm{pred,j}}-y_{\mathrm{actual,j}}\right)}^{2}},$$ (4)

where *n* is the number of observations, *y*_*pred,j*_ is the predicted value for sample *j*, and *y*_*actual,j*_ is the actual value for sample *j*.

To further quantify the relative performance of different normalization methods, we ranked all normalization methods based on the median RMSE values when the model was trained and validated on the same pair of training and testing datasets. The median RMSE values were arranged in ascending order with ranks ranging from one to 22. For a given method, a lower median RMSE corresponded to a lower ranking value, which indicated better relative performance among the 22 normalization methods being compared.

## 3 Results

### 3.1 The reproducibility of cross-study prediction is limited by various confounding variables

Using the inclusion criteria outlined in Section 2.2, we incorporated a total of 5,963 healthy stool samples into our analysis. These samples were obtained from 31 different datasets and exhibited various biological and technical differences, encompassing variations in geographic origin, DNA extraction techniques, and sequencing platforms (Table 1).

Initially, we examined the BMI values and Shannon indices within each dataset to identify the overall patterns in sample characteristics. Supplementary Figure S1 demonstrates noticeable differences in BMI among samples from different datasets, with each dataset having its own distinct range. The average BMI values for each dataset varied significantly, ranging from 21.2 (DhakanDB_2019) to 30.7 (KeohaneDM_2020), while the overall average BMI for all samples was 24.9. To assess the significance of these variations, we performed Wilcoxon tests comparing the BMI of each dataset with the overall sample mean. Among the 31 datasets, 10 displayed significantly higher BMIs than the overall sample mean, while 11 exhibited significantly lower BMIs. The Shannon indices, as presented in Supplementary Figure S2, generally mirrored the trends observed in BMI values, although differences persisted. Notably, KeohaneDM_2020, despite having the highest average BMI, demonstrated significantly lower Shannon indices compared to the overall dataset. Conversely, LeChatelierE_2013 displayed a significantly higher average BMI than the overall average but exhibited no significant differences in Shannon indices.

The similarities among different datasets were evaluated by PCoA plot based on the Bray-Curtis distance, as depicted in Figure 2A. Owing to the large sample sizes, the mean point of each dataset was used to represent the positions of the samples from that dataset on the PCoA plot. Additionally, the size of the points indicated the sample size of each dataset. This plot revealed distinct separations between the datasets, indicating variations in microbial composition. To further assess the contribution of biological or technical factors to microbiome variation, PERMANOVA was performed on the Bray-Curtis distance. Figure 2B illustrates that all seven factors considered in the analysis accounted for a significant proportion of the variations. The three most influential factors affecting the community structures of the microbiome data were datasets, country, and DNA extraction kit, followed by sequencing platform, age, gender, and BMI.

Subsequently, the impact of heterogeneities on the reproducibility of BMI prediction was examined based on abundance profiles normalized by the simplest the most commonly used method, TSS. The classifier was trained on each individual training set (dataset 1), and the model was applied to each testing set (dataset 2). The cross-prediction matrix of RMSE values, obtained using random forest models on TSS normalized abundances, is illustrated in Figure 2C. The median RMSE values exhibited variation across datasets and were influenced by multiple factors. Importantly, a significant disparity in RMSE values was observed when different datasets were used as the training dataset to predict KeohaneDM_2020. Specifically, 18 out of 30 datasets exhibited RMSE values exceeding 7, indicating an inaccurate prediction of the BMI in KeohaneDM_2020. This finding aligned with the substantial differences in microbiome composition compared to the other datasets. LeChatelierE_2013 was another dataset demonstrating relatively poor prediction repeatability, despite not differing significantly from other datasets in terms of its Shannon indices. In contrast, the dataset LifeLinesDeep_2016 displays significant differences in the PCoA plot compared to other datasets, yet it performs well in predicting and can also be effectively predicted by other datasets. This phenomenon can possibly be attributed to its relatively larger sample size. In conclusion, these results indicate that the reproducibility of response predictions were influenced by various factors.

The presence of confounding factors, such as country and DNA extraction kit, led to notable variations in the background distributions of taxa. We conducted an evaluation to ascertain whether models trained on one dataset could accurately predict a quantitative phenotype for samples in another dataset. Additionally, we examined whether the implementation of normalization methods could enhance prediction performance in the following sections.

### 3.2 Batch correction methods are necessary for quantitative phenotype prediction

In our simulation studies, we evaluate the influence of heterogeneity on prediction performance across three distinct scenarios and using four different types of quantitative phenotypes. Additionally, we analyze and compare the prediction performance of various normalization methods.

In Scenario 1, we evaluated the impact of various normalization methods on the prediction of quantitative phenotypes across diverse background distributions of taxa. The experiments were repeated 100 times, and the median RMSE values were calculated based on abundance profiles normalized by different methods. The results are presented in Figure 3. We find that prediction accuracy decreases as population effects increase, as evident from the corresponding increase in RMSE values. However, different normalization methods exhibit minimal variation in predicting quantitative phenotypes. For instance, considering the linear relationship of phenotypes (Figure 3A), at a population effect of 0.2, the maximum and minimum RMSE values for different methods differ by 0.08. This difference slightly increases with an increase in population effect, peaking at a population effect of 1, where it reaches 0.23—still a very small difference. Similar trends are observed for quadratic (Figure 3B), inverse (Figure 3C), and logistic (Figure 3D) relationships of phenotypes. These findings suggest that, among the 22 normalization methods we compared, none of them outperforms the others in predicting quantitative phenotypes when the population effect is fixed. We further rank the median RMSE of different normalization methods derived from the identical set of simulation parameters, such as a linear relationship and ep = 0. The distribution of rankings can be found in Supplementary Figure S3A. Despite observing variations in the relative performance of different methods, the minor disparities in median RMSE render little reference values for rankings in Scenario 1.

In Scenario 2, we investigated the impact of batch effects on model prediction performance when utilizing abundance profiles normalized by different methods. Figure 4 demonstrates the median RMSE obtained from random forest models using abundance profiles normalized by different methods across 100 runs. As expected, we observe an increasing trend in RMSE values for all methods as batch effects increase. Interestingly, we find that batch effects of the mean taxa abundance appeared to have a greater impact than the variance of taxa abundance, especially in terms of model prediction performance. Furthermore, when compared to scaling methods and transformation methods, batch correction methods exhibit lower RMSE values, particularly when large batch mean differences are present. This trends are also validated by the lower ranks of batch correction methods (Supplementary Figure S3B). Among the six batch correction methods, in the case of a logistic relationship of phenotypes (Figure 4D), when the batch variance is set to one and the batch mean to 500 or 1,000, BMC and Limma demonstrate similar performance, with the lowest predictive accuracy among all normalization methods. However, their respective RMSE values differ by no more than 0.05 from the minimum RMSE value. This minimal difference can be considered negligible in practical predictions. Additionally, the performance of ComBat is noteworthy. It exhibits lower predictive accuracy than other batch correction methods in linear (Figure 4A), quadratic (Figure 4B), and inverse (Figure 4C) relationships of phenotypes. However, in the case of a logistic relationship of phenotypes, it outperforms all other methods. This inconsistency highlights the need for caution when using ComBat for batch correction.

**FIGURE 5:**
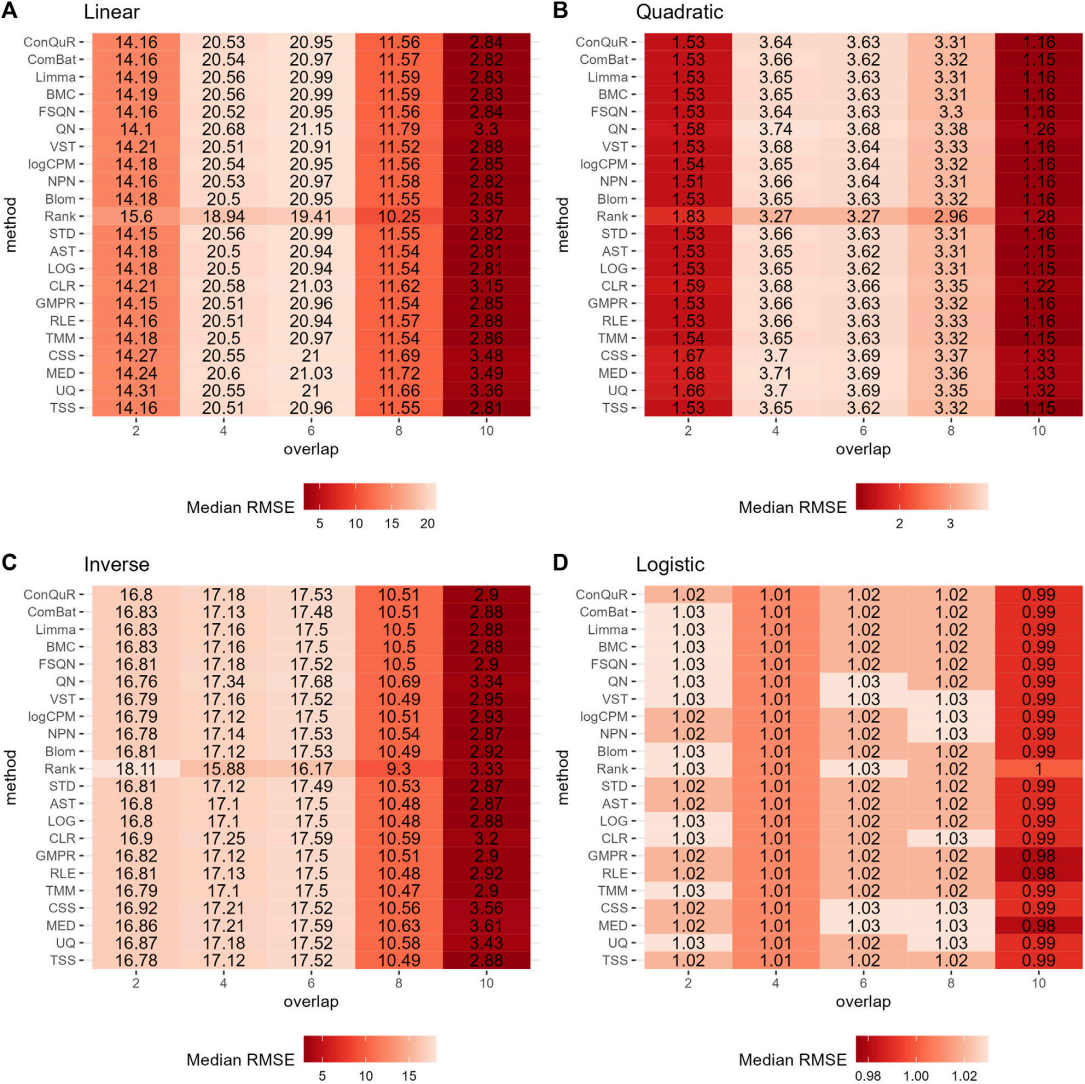
Heatmaps depicting median RMSE values obtained from abundance profiles normalized by different methods for predicting simulated quantitative phenotype in Scenario 3. The panels correspond to relationships between phenotype and phenotype-associated taxa, including **(A)** linear, **(B)** quadratic, **(C)** inverse, and **(D)** logistic. The columns represent different numbers of overlapping disease-associated taxa in the training and testing datasets. The rows represent different normalization methods.

Figure 5 illustrates the findings from simulation scenario 3, which investigated the influence of different phenotype-associated feature models. Ideally, as the number of overlapping phenotype-associated taxa increases, the RMSE values should decrease. However, the choice of these taxa can significantly impact the prediction of quantitative phenotypes due to the shared background distributions of taxa. If randomly selected phenotype-associated taxa predominantly have zero values, it leads to similarity in the phenotype model during training and testing. This phenomenon is particularly noticeable when the overlapping number is two in both linear (Figure 5A) and quadratic (Figure 5B) relationships of phenotypes. In these cases, the median RMSE value at overlap = 2 is lower than the median RMSE value at overlap = 4. Across the four different types of quantitative phenotypes, the RMSE reaches its minimum at overlap = 10, suggesting that at this point, phenotypes can be accurately predicted. However, similar to scenario 1, the performance of different normalization methods remains relatively consistent, with no single method significantly outperforming the others in predicting quantitative phenotypes. Moreover, the rankings of different normalization methods in Supplementary Figure S3C do not possess any meaningful reference value due to the negligible differences among them in the same parameter combinations.

In our simulations for predicting quantitative phenotypes, we consistently found that no normalization method exhibited consistent advantages over the others. However, given the frequent occurrence of batch effects and the satisfactory performance of batch correction methods in predicting datasets with such effects in both the training and testing sets, we highly recommend utilizing batch correction methods as an initial step prior to predicting quantitative phenotypes.

### 3.3 Use QN and ComBat normalization carefully in quantitative phenotype prediction

In the following analysis, we assessed the performance of different normalization methods using a set of 31 shotgun sequencing datasets obtained from healthy stool samples (Table 1). Each dataset was paired, with one assigned for model training and the other for validation purposes. For each method, we calculated the RMSE values based on the normalized abundance using a random forest model. To account for the randomness inherent in the prediction model, we repeated the predictions 10 times and report the median RMSE value for each study.

Supplementary Figure S4 shows boxplots of the median RMSE obtained from predictions made using various models on specific test datasets. Within these specific test datasets, we performed Wilcoxon tests to evaluate the differences in means between different methods and the average mean. Our observations indicate that all methods encounter limitations due to biological and technical factors when predicting quantitative phenotypes, despite their best efforts. None of the methods exhibited significant reductions in the prediction’s RMSE, and no significant differences were observed among them. This aligns with the conclusions derived from our simulations. For instance, as shown in Supplementary Figure S4C2, when KeohaneDM_2020 was used as the test dataset while others served as training sets, the RMSE values varied from 5.5 to 11.9 depending on the selected training data. The median RMSE values were approximately 8.2, without any significant differences observed among them.

**FIGURE 6:**
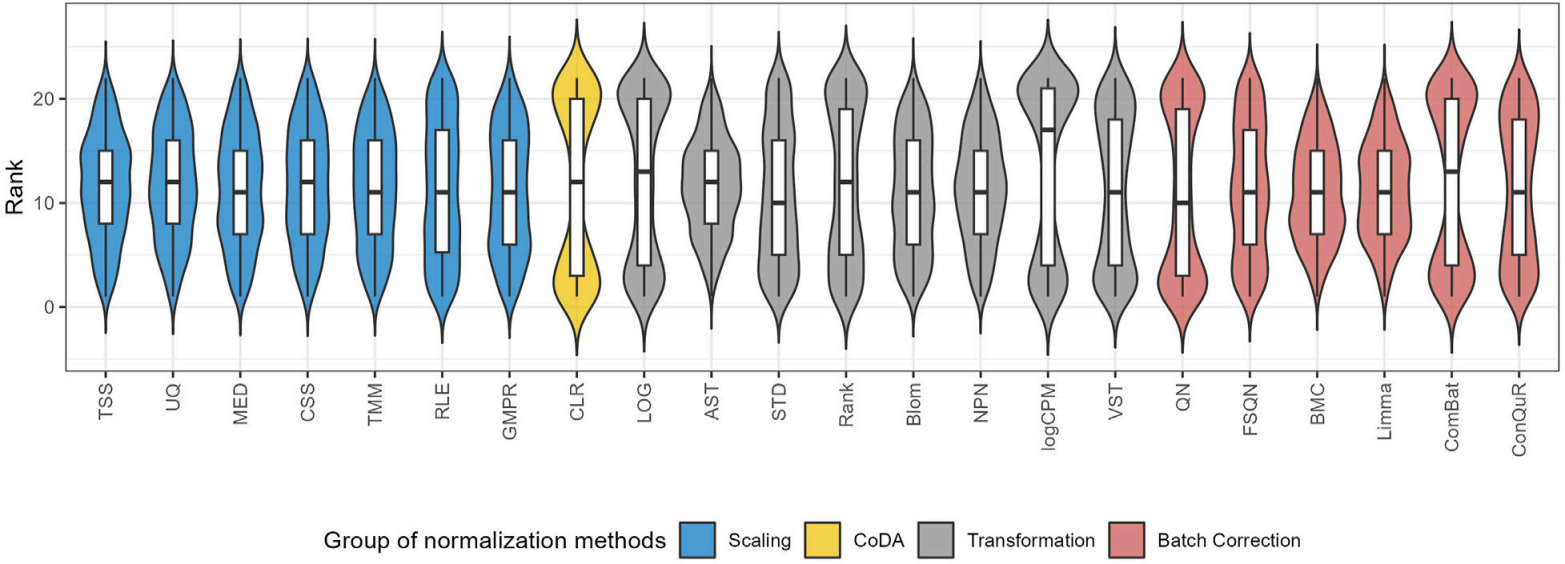
The distribution of the ranks for different normalization methods according to the median RMSE value under the same pair of training and testing datasets.

To quantify the performance of normalization methods, we ranked all normalization methods based on the median RMSE values when the model was trained and validated on the same pair of training and testing datasets. Figure 6 shows the distributions of the ranks for each method across the 31 studies. A higher ranking (lower values in the box plot) indicates a better prediction performance. While all normalization methods had similar performance, batch correction methods exhibited slightly better results. It is worth mentioning that QN and ComBat, among the batch correction methods, displayed fluctuations that made them susceptible to extreme rankings compared to the other twenty-two normalization methods. Methods like CLR, LOG, and logCPM showed similar patterns. Therefore, caution should be exercised when employing these methods. Based on these findings, we suggest employing batch correction methods like FSQN, BMC, and Limma when making predictions for quantitative phenotypes.

## 4 Discussion

Normalization is an essential step in metagenomic data analysis. Various normalization methods have been proposed to mitigate the challenges of heterogeneity. While the comparison of these methods focused on their impact on differential analysis (McMurdie and Holmes, 2014; Weiss et al., 2017), the effects of heterigeneity on quantitative phennotype predictions have not been well addressed. Therefore, in this study, we investigated the influence of different normalization methods in cross-datasets prediction of quantitative phenotypes using simulations and real heterogeneous datasets.

In our simulations, we examined three sources of heterogeneity: population effects, batch effects, and phenotype-associated feature models. Population effects account for variations resulting from differences in population characteristics, including environmental factors, geographical locations, and others. Batch effects arise from technical variations introduced during data collection or processing, such as sequencing technologies, sample preparation, or experimental procedures. Phenotype-associated feature models represent the underlying patterns and features associated with the targeted phenotype. Discrepancies in this aspect can lead to reduced predictive performance, as a model trained on one dataset may struggle to effectively generalize to another dataset.

Furthermore, we examined four types of relationships between microbial features and quantitative phenotypes in the simulations, including linear, quadratic, inverse, and logistic. A linear relationship represents the simplest connection between microbial features and quantitative phenotypes. In this type of relationship, changes in microbial features result in proportional changes in quantitative phenotypes. For instance, Turnbaugh et al. demonstrated a linear-like relationship between the abundance of Bifidobacteria and the concentration of fecal short-chain fatty acids (SCFA) (Turnbaugh et al., 2006). However, the human microbiome is a complex ecosystem, leading to non-linear interactions between microbial features and phenotypes. We considered three non-linear relationships: quadratic, inverse, and logistic. A quadratic relationship suggests that changes in phenotypes initially occur proportionally but deviate beyond a certain threshold, resulting in a curved or parabolic shape. For example, Akkermansia muciniphila has been associated with improvements in metabolic parameters such as insulin sensitivity and lipid profile. However, both low and excessively high levels of Akkermansia muciniphila are linked to metabolic dysfunction, indicating a non-linear relationship with metabolic phenotype (Dao et al., 2016). An inverse relationship indicates competitive or inhibitory interactions between microbial taxa or functional groups where an increase in one taxon leads to a decrease in another, resulting in reciprocal changes in phenotype levels. For example, *Streptococcus* mutans negatively correlates with dental caries susceptibility, where higher levels of *Streptococcus* mutans increase the risk of caries development (Takahashi and Nyvad, 2011). A logistic relationship describes a sigmoidal curve where changes in one variable initially have minimal effects on the phenotype, followed by a rapid increase or decrease until reaching a plateau or asymptote. This relationship can be observed in *E. coli* and urinary tract infection (UTI) severity (Kaper et al., 2004). In low abundance, *Escherichia coli* may act as a commensal or beneficial organism in the gut microbiota. However, beyond a certain threshold, colonization of uropathogenic *E. coli* in the urinary tract can lead to UTIs, with increasing abundance correlating with higher UTI severity, illustrating a logistic relationship between *E. coli* abundance and UTI risk. Together with the real heterogeneous datasets, our investigation provides comprehensive insights into the performance and suitability of different normalization approaches for predicting quantitative phenotypes.

In our previous study of binary phenotype prediction (Wang et al., 2024), scaling methods, such as TMM, demonstrated relatively good performance, while transformation methods, including NPN and Blom, exhibited promising results in certain datasets. Furthermore, batch correction methods, such as BMC and Limma, consistently performed well across multiple datasets. The challenges encountered in predicting quantitative phenotypes were evident across all normalization methods, as none of them achieved a significant reduction in the RMSE of the predictions irrespective of the approach employed. These findings align with our simulations and underscore the complex nature of metagenomic data, which is prone to both biological and technical variations. Hence, it is reasonable to infer that the limitations are inherent to the data itself rather than being contingent on the choice of normalization method.

The absence of significant differences among the normalization methods is an important observation. Despite considering a wide range of relationships between phenotypes and taxa abundance profiles (linear, quadratic, inverse, and logistic), the variation in RMSE values remains consistently low across the methods. This performance of normalization methods across various scenarios is a valuable finding as it enables researchers to choose methods based on other criteria without compromising predictive performance.

However, our analysis unveiled a modest advantage of batch correction methods over other normalization techniques. Specifically, we observed slightly improved results when employing these methods. Among the recommended methods for predicting quantitative phenotypes are FSQN, BMC, and Limma. Although they may not yield drastic performance enhancements, their slightly superior performance signifies potential robustness in tackling the inherent challenges of metagenomic data, particularly in the prediction of quantitative phenotypes.

It is crucial to acknowledge that certain normalization methods, namely, QN and ComBat, exhibited fluctuations that heightened their susceptibility to extreme rankings. These fluctuations underscore the importance of exercising caution when selecting specific normalization techniques. Hence, researchers must carefully evaluate the suitability of a chosen method for their particular dataset and research question, taking into account the unique characteristics and potential fluctuations inherent in their data. Unfortunately, the heterogeneity between datasets is the result of multiple factors, and our current data does not support the selection of unstable methods like QN or ComBat. Further research is needed to quantify the magnitude of heterogeneity and its impact on predictions. As a result, we did not recommend methods with extreme rankings, such as QN and ComBat.

In conclusion, the performance of normalization methods in analyzing metagenomic data remains an active and ongoing area of research. Our study contributes to this field by conducting a comprehensive evaluation of various methods and providing valuable insights into their effectiveness in predicting quantitative phenotypes. From our findings, it appears that batch correction methods may be preferable. However, it is still crucial for researchers to continue exploring and developing novel techniques to further enhance the accuracy of predictions in the intricate realm of metagenomic data. Ultimately, the selection of a normalization method should be made judiciously, considering the specific characteristics of the dataset and the research objectives, as there is currently no universally applicable solution in this challenging domain. If there is limited knowledge about the datasets, we recommend incorporating batch correction methods, such as BMC or Limma, into the quantitative phenotype prediction of metagenomic data across different datasets. This involves using scaling methods to reduce biases introduced by sequencing technology, and then applying a LOG transformation to approximate a more normally distributed data, aligning with the assumptions of batch correction methods. By subsequently employing batch correction methods, we enhance the robustness of the analysis. We posit that this pipeline has the potential to improve the accuracy and reliability of cross-dataset predictions of quantitative phenotypes based on metagenomic data.

## Data Availability

The original contributions presented in the study are included in the article/Supplementary Material, further inquiries can be directed to the corresponding author. All the datasets used in this study are available in the R package curated Metagenomic Data (v3.8.0). All the codes used in the analysis can be found at https://github.com/wbb121/NormMethodsComp--QuantPred.
