# Supplementary material for: Evaluation of normalization methods for predicting quantitative phenotypes in metagenomic data analysis
Source: Front Genet. 2024 Jun 5;15:1369628. doi: 10.3389/fgene.2024.1369628 (PMC11188486; doi:10.3389/fgene.2024.1369628)
Supplement: Supplementary file 1 [file DataSheet1.PDF]

Supplementary Material

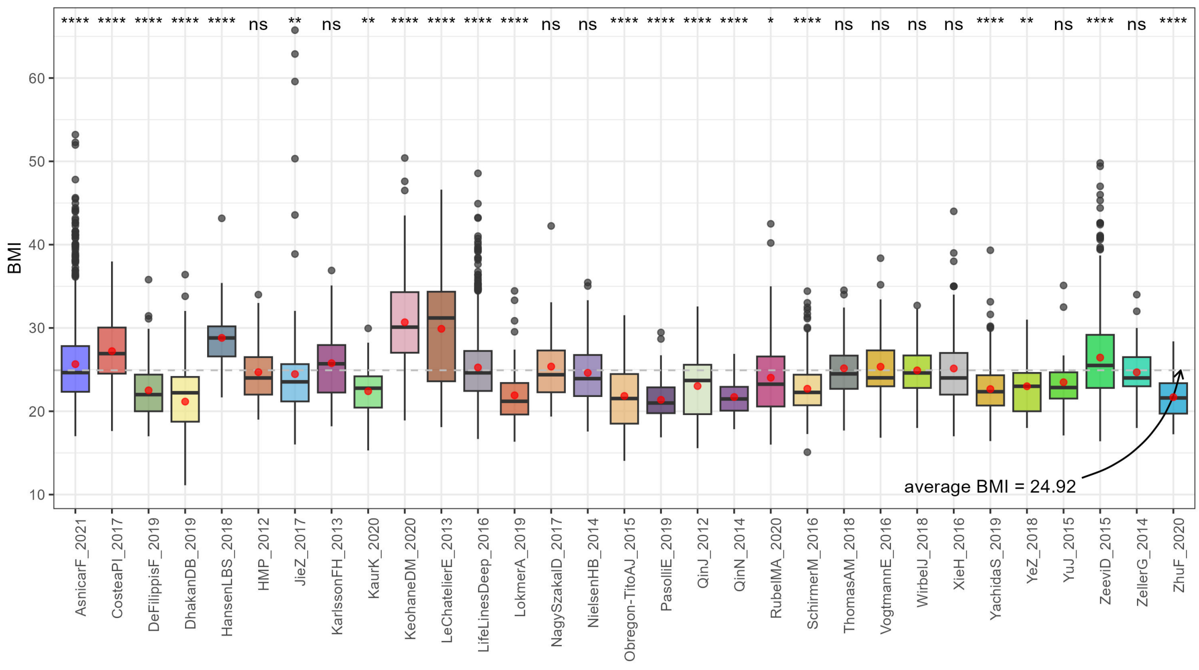

Figure S1. Boxplot illustrating the distribution of BMI values across different datasets. The red dots indicate the average BMI for each dataset, and the gray dashed line represents the overall BMI mean. The significance of the difference between dataset BMIs and the overall BMI was determined using the Wilcoxon test, where ns signifies non-significance, * denotes a p-value < 0.05, ** indicates p-value < 0.01, *** suggests p-value < 0.001, and **** represents p-value < 0.0001.

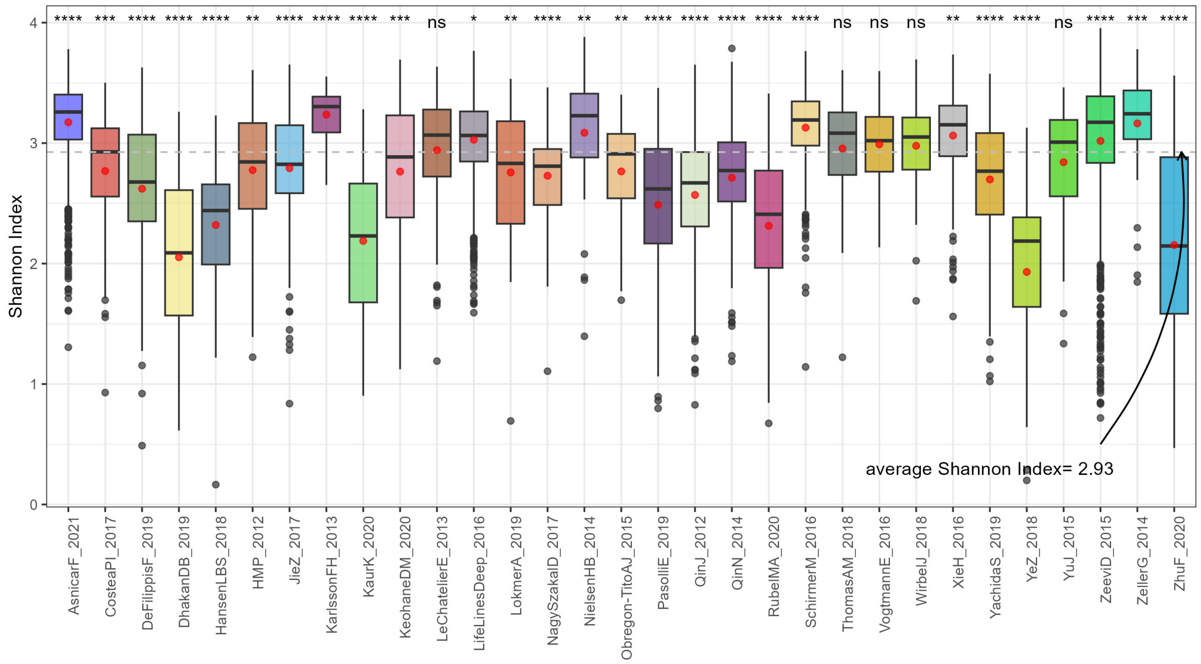

Figure S2. Boxplot illustrating the distribution of Shannon Index across different datasets. The red dots indicate the average Shannon Index for each dataset, and the gray dashed line represents the overall average Shannon Index. The significance of the difference between dataset Shannon Indices and the overall Shannon Indices was determined using the Wilcoxon test, where ns signifies non-significance, * denotes a p-value < 0.05, ** indicates p-value < 0.01, *** suggests p-value < 0.001, and **** represents p-value < 0.0001.

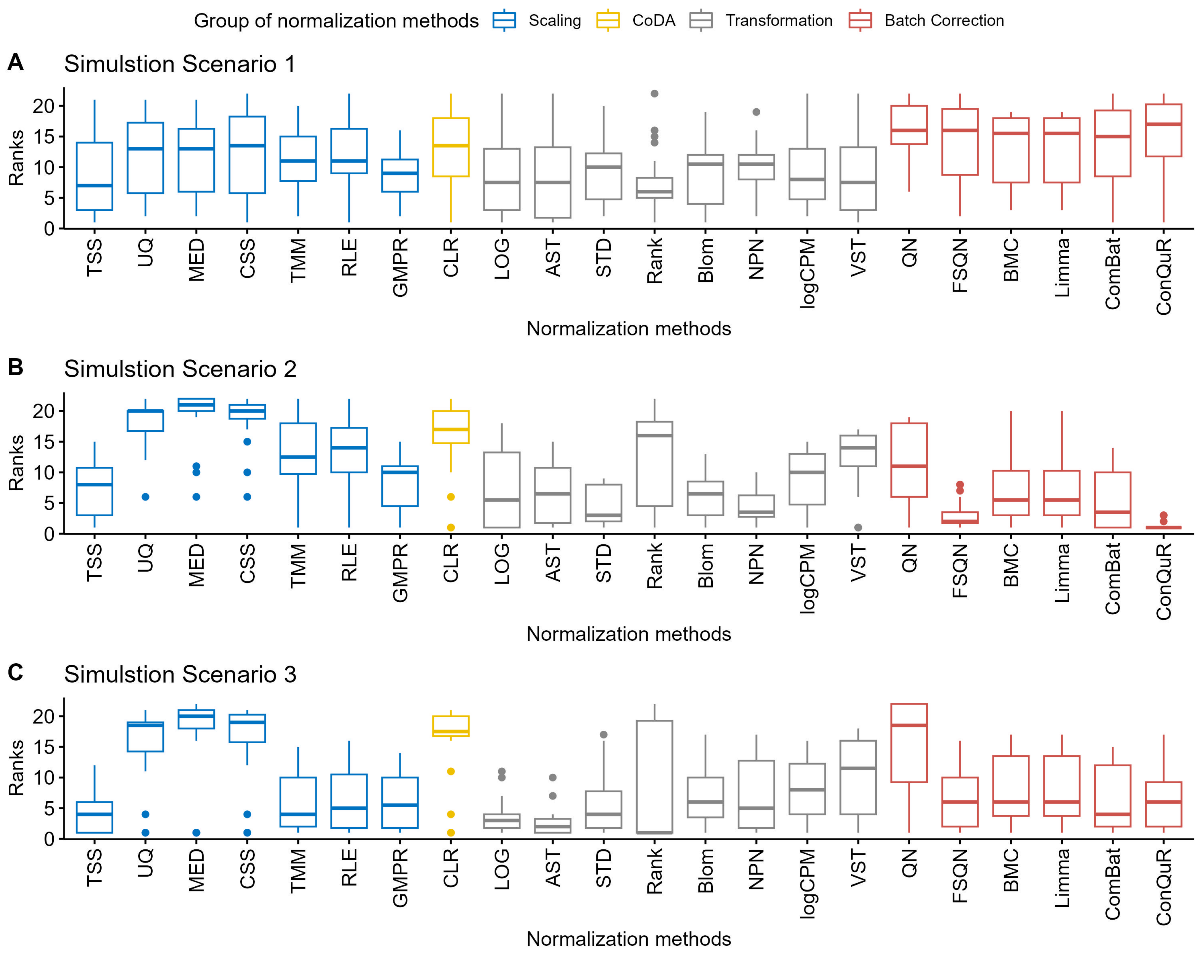

Figure S3. Predictive performance of normalization methods in different simulation scenarios. The normalization methods are ranked based on the median RMSE under the same combination of simulation parameters.

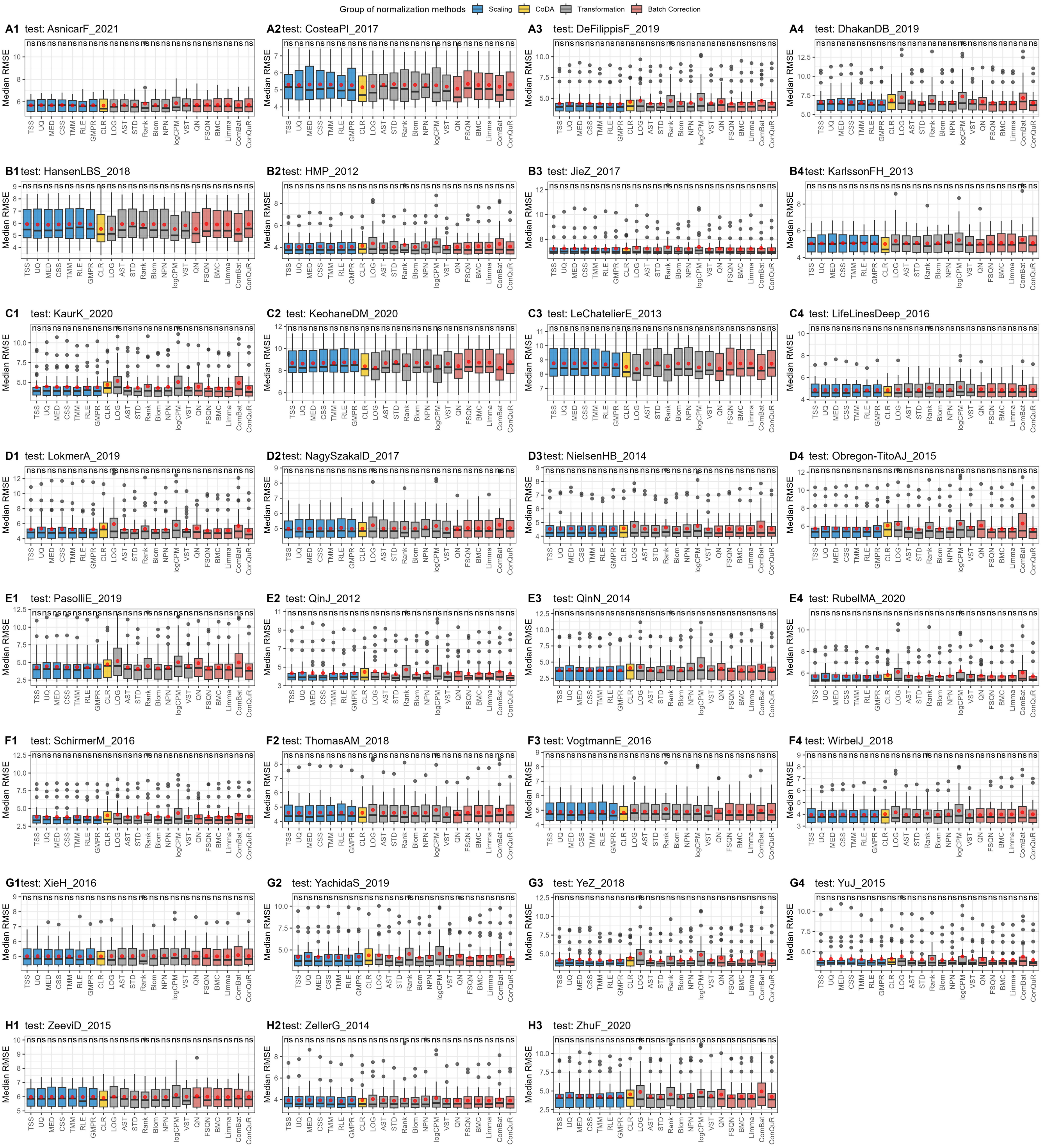

Figure S4. Predictive performance of different normalization methods using 31 healthy stool datasets from curatedMetagenomicData. This figure comprises a collection of boxplots, each representing the predictive performance of different normalization methods in a specific test dataset. The x-axis enumerates various normalization techniques, while the y-axis indicates the median RMSE obtained from ten repeated predictions. The red dots signify the mean median RMSE for each method. The significance of differences between the method means and the overall mean was assessed by the Wilcoxon test, with ”ns” for non-significant results.
